# TF-EPI: an interpretable enhancer-promoter interaction detection method based on Transformer

**DOI:** 10.3389/fgene.2024.1444459

**Published:** 2024-08-09

**Authors:** Bowen Liu, Weihang Zhang, Xin Zeng, Martin Loza, Sung-Joon Park, Kenta Nakai

**Affiliations:** ^1^ Department of Computational Biology and Medical Sciences, Graduate School of Frontier Sciences, University of Tokyo, Tokyo, Japan; ^2^ Human Genome Center, Institute of Medical Science, University of Tokyo, Tokyo, Japan

**Keywords:** Transformer, enhancer-promoter interactions, motif discovery, attention mechanism, transfer learning

## Abstract

The detection of enhancer-promoter interactions (EPIs) is crucial for understanding gene expression regulation, disease mechanisms, and more. In this study, we developed TF-EPI, a deep learning model based on Transformer designed to detect these interactions solely from DNA sequences. The performance of TF-EPI surpassed that of other state-of-the-art methods on multiple benchmark datasets. Importantly, by utilizing the attention mechanism of the Transformer, we identified distinct cell type-specific motifs and sequences in enhancers and promoters, which were validated against databases such as JASPAR and UniBind, highlighting the potential of our method in discovering new biological insights. Moreover, our analysis of the transcription factors (TFs) corresponding to these motifs and short sequence pairs revealed the heterogeneity and commonality of gene regulatory mechanisms and demonstrated the ability to identify TFs relevant to the source information of the cell line. Finally, the introduction of transfer learning can mitigate the challenges posed by cell type-specific gene regulation, yielding enhanced accuracy in cross-cell line EPI detection. Overall, our work unveils important sequence information for the investigation of enhancer-promoter pairs based on the attention mechanism of the Transformer, providing an important milestone in the investigation of cis-regulatory grammar.

## 1 Introduction

In the three-dimensional (3D) space of the cell nucleus, enhancers are crucial for cells to effectively process genetic information. As key regulatory DNA elements, enhancers influence cell functions by establishing physical contacts with their target-gene promoters, which sometimes span substantial genomic distances (Pombo and Dillon, 2015; Furlong and Levine, 2018; Schoenfelder and Fraser, 2019). Interactions between enhancers and promoters have thus, become a key area of research. These interactions are not only vital for gene initiation and regulation but also offer insights into how the 3D organization of DNA within the nucleus impacts the way cells acquire and interpret genetic information.

Enhancer–promoter interactions (EPIs) can be discovered using many high-throughput sequencing techniques, including paired-end tag sequencing (ChIA-PET) (Fullwood et al., 2009) and high-throughput chromosome conformation capture (Hi-C) (Rao et al., 2014). For instance, multiple topologically associating domains (TADs) can be discovered by Hi-C, and the mammalian chromatin interaction frequency has been proven to be much higher inside than outside such TADs (Dixon et al., 2012). Another novel method to study interactions is the promoter capture Hi-C (PCHi-C) technique, which focuses on interactions in promoter regions by directly targeting them with high precision (Schoenfelder et al., 2018). Although these sequencing methods have demonstrated a strong capability to detect EPIs, they are limited for various reasons. For example, PCHi-C requires deep sequencing to achieve high spatial resolution for examining chromatin interactions centered on promoters, which is often time-consuming and expensive.

With the advances in machine learning and deep learning techniques, numerous computational methods have been developed to predict EPIs. These methods typically use genomic and epigenomic signals or sequence information, often extracted from enhancer and promoter regions or their adjacent extended genomic regions. Methods such as RIPPLE (Roy et al., 2015), TargetFinder (Whalen et al., 2016), and TransEPI (Chen et al., 2022) use functional genomics data like ChIP-seq and DNase-seq data to generate such features. Subsequently, using machine learning or deep learning methods, these features are categorized to obtain the final EPI classification results. These epigenomic signal-based methods have achieved relatively accurate prediction capacities. However, they often require the incorporation of vast amounts of functional genomics data to provide sufficient feature information. This introduces certain constraints when these methods are applied to new samples, that lack certain specific types of sequencing data.

On the other hand, the methods like EPIANN (Mao et al., 2017), SPEID (Singh et al., 2019), and EPI-DLMH (Min et al., 2021) extract features directly from DNA sequences without requiring additional features. Although these methods address the complexity of the data required by the aforementioned epigenomic signal-based methods, their implementation is limited, e.g., focusing solely on the dataset of TargetFinder. This dataset has certain issues, such as suffering from inflated performance evaluation (Xi and Beer, 2018). Moreover, since these methods commonly employ convolutional neural networks (CNN) for classification, it is challenging to pinpoint which specific sub-sequences of enhancers and promoters play a decisive role in the classification results, leading to limited model interpretability.

In addition, other methods such as DeepC (Schwessinger et al., 2020), Akita (Fudenberg et al., 2020), ChiNN (Cao et al., 2021) and Enformer (Avsec et al., 2021) have demonstrated the capability to explore chromatin interactions related to different types of data, particularly Hi-C data. Nevertheless, they also have certain limitations: DeepC, Akita and Enformer require constraining of the input sequences within a certain distance to detect chromatin interactions, which introduce unnecessary computation and prevents the model from detecting EPIs that exceed sequence length limits. ChiNN also suffers from reduced interpretability due to its use of a CNN model with fixed parameters for classification, making it unable to assess the contribution of different parts of the sequence to the final classification outcome.

In recent years, Transformer (Vaswani et al., 2017) has emerged as a significant model architecture in the field of deep learning for natural language processing and DNA sequence analysis (Ji et al., 2021). In this study, given the limitations of the existing methods, we developed a Transformer-based EPI prediction method called TF-EPI, which requires only DNA sequences as input, enabling our method to overcome data input limitations. We extensively validated the accuracy of our model using multiple benchmark datasets, including cross-cell line EPI prediction. We demonstrate how the Transformer helps the model to obtain comprehensive sequence information and how we can utilize its attention mechanism to provide sequence-level interpretable predictions.

## 2 Materials and methods

### 2.1 Preparing datasets

Two publicly available EPI datasets were prepared (Supplementary Note S1; Supplementary Table S1); the Benchmark of Candidate Enhancer-Gene Interactions (BENGI) dataset (Moore et al., 2020), including 6 cell lines (GM12878, HeLa-S3, HMEC, IMR90, K562, and NHEK), and K562 in the Fulco dataset compiled with CRISPR validated EPIs (Nasser et al., 2021). Similarly, in each cell line of these datasets, the number of negative enhancer-promoter pairs significantly exceeded that of the positive ones. For all cell lines, we set the promoter and enhancer sequences as 2k-bps (1,500 bp upstream and 500 bp downstream of the transcription start site, TSS) and 3k-bps, respectively, as widely used in previous studies (Mao et al., 2017; Singh et al., 2019; Min et al., 2021).

For each cell line, we generated three separate training, validation and test sets. Given that the data were severely imbalanced across classes, we used data augmentation techniques common to both the EPIANN and EPI-DLMH for a fair comparison, adjusting the training set to balance the number of positive and negative samples (the specific methods are detailed in the Supplementary Material).

### 2.2 Tokenizer

We used a k-mer representation to tokenize each DNA sequence of the enhancers and promoters. In this study, we set k to 6, as this has been extensively used in several studies based on DNA sequences (Ji et al., 2021; Min et al., 2021), and it helps the model to find longer motifs during downstream analysis. For instance, a given DNA sequence “TCGTCACT” can be tokenized to a sequence of three 6-mers: TCGTCA, CGTCAC, and GTCACT. We added a special token “CLS” at the beginning of each set of 6-mers to assist with the classification task. In addition, we inserted a special token “SEP” between the enhancer tokens and promoter tokens to separate the two sequence sections.

### 2.3 Model pre-training

To pre-train our model, we selected non-overlapping DNA sequences of lengths no longer than 5,000 bp from the human reference genome hg19. Subsequently, for each sequence, we masked 15 percent of its tokens, and trained the model until convergence. For a more detailed description of the pre-training process, please refer to the Supplementary Material.

### 2.4 Model fine-tuning

We used our pre-trained model for subsequent TextCNN classification utilizing cell type-specific data. The TextCNN includes a set of three convolutional kernels with six dimensions. After the convolutional layer, the obtained feature vectors undergo max pooling, and the integrated result is fed into a fully connected layer to obtain the classification outcome. For more detailed information on the fine-tuning process, please refer to Supplementary Note S2.

### 2.5 *De novo* motif discovery

We averaged the attention matrices across different heads from the last Transformer encoder layer. We then computed the average attention of each 6-mer towards all tokens, decomposed the attention of 6-mer into the attention of each individual base and normalized the overall average values as follows:

$${attention}_{i}^{normalized}=\frac{{attention}_{i}^{mean}-{attention}^{\min}}{{attention}^{\max}}$$

where ${attention}^{\max}$ and ${attention}^{\min}$ represent the maximum and minimum among all the ${attention}_{i}^{mean}$ , respectively, for all bases of each enhancer and promoter (EP) pair.

We selected high attention regions based on the normalized mean attention values of each base. As in DNABERT, for each high attention region, we found significantly enriched short sequences in the positive sequences based on the hypergeometric test, as follows:

$$P\left({k-mer}_{pos}=k\right)=\frac{\left(\begin{array}{l} K\\ k \end{array}\right)\cdot \left(\begin{array}{l} N-K\\ n-k \end{array}\right)}{\left(\begin{array}{l} N\\ n \end{array}\right)}$$

where *k* represents the number of positive EP pairs containing a specific high attention sequence. *N* represents the total number of EP pairs, *K* represents the number of all positive EP pairs, and *n* represents the number of EP pairs containing a specific high attention sequence among all EP pairs. Finally, we filtered out the short sequences with high confidence based on the *p*-value of the hypergeometric test and merged them using method identical to that employed in DNABERT to produce the final motif sequence.

### 2.6 Motif comparison

Each discovered motif was matched with known human motifs in the JASPAR database using Tomtom (Gupta et al., 2007; Fornes et al., 2020). UniBind is another database that uses ChIP-seq data to predict transcription factor binding sites in different cell lines (Gheorghe et al., 2019; Puig et al., 2021). This database was used to verify whether these motifs were biologically significant.

### 2.7 Cross-cell type EPI detection model

We introduced a domain-adaptive neural network (DANN) based on transfer learning (Ganin and Lempitsky, 2015) to detect cross-cell type EPIs. Similar to our cell type-specific EPIs, we used an additional classifier to determine whether the input sample originated from the source or the target cell line. During the training process, the embedding output from the final Transformer encoder layer passes through a gradient reversal layer (GRL). In forward propagation, the GRL does not alter its input; however, during backward propagation, it inverts the gradient (Ganin and Lempitsky, 2015).

The loss function of the entire network consists of two parts: the classification loss function, which is used to classify labeled data in the source cell line, and the domain loss function, which makes feature representations from the source and target cell lines indistinguishable.

$$L_{class}=-\sum_{i\epsilon src}y_{i}\log\;\left(p\left(y_{i}|x_{i}\right)\right)$$

$$L_{domian}=-\sum_{i\epsilon src}\log\left(p\left(domain=src|x_{i}\right)\right)-\sum_{j\epsilon tgt}\log\;\left(p\left(domain=\left.tgt\right|x_{j}\right)\right)$$

where $x_{i}$ and $x_{j}$ are the samples from the source and target cell lines, respectively, and the $p\left(domain=src|x_{i}\right)$ and $p\left(domain=\left.tgt\right|x_{j}\right)$ are the domain distributions predicted by the model.

The overall loss function is a combination of these two losses:

$$L_{total}=L_{class}-{\lambda L}_{domian}$$

The λ is used to balance the two losses. In this way, the model can focus both on the feature expression of all data and the classification tasks.

## 3 Results

### 3.1 Overall structure of TF-EPI

**FIGURE 1:**
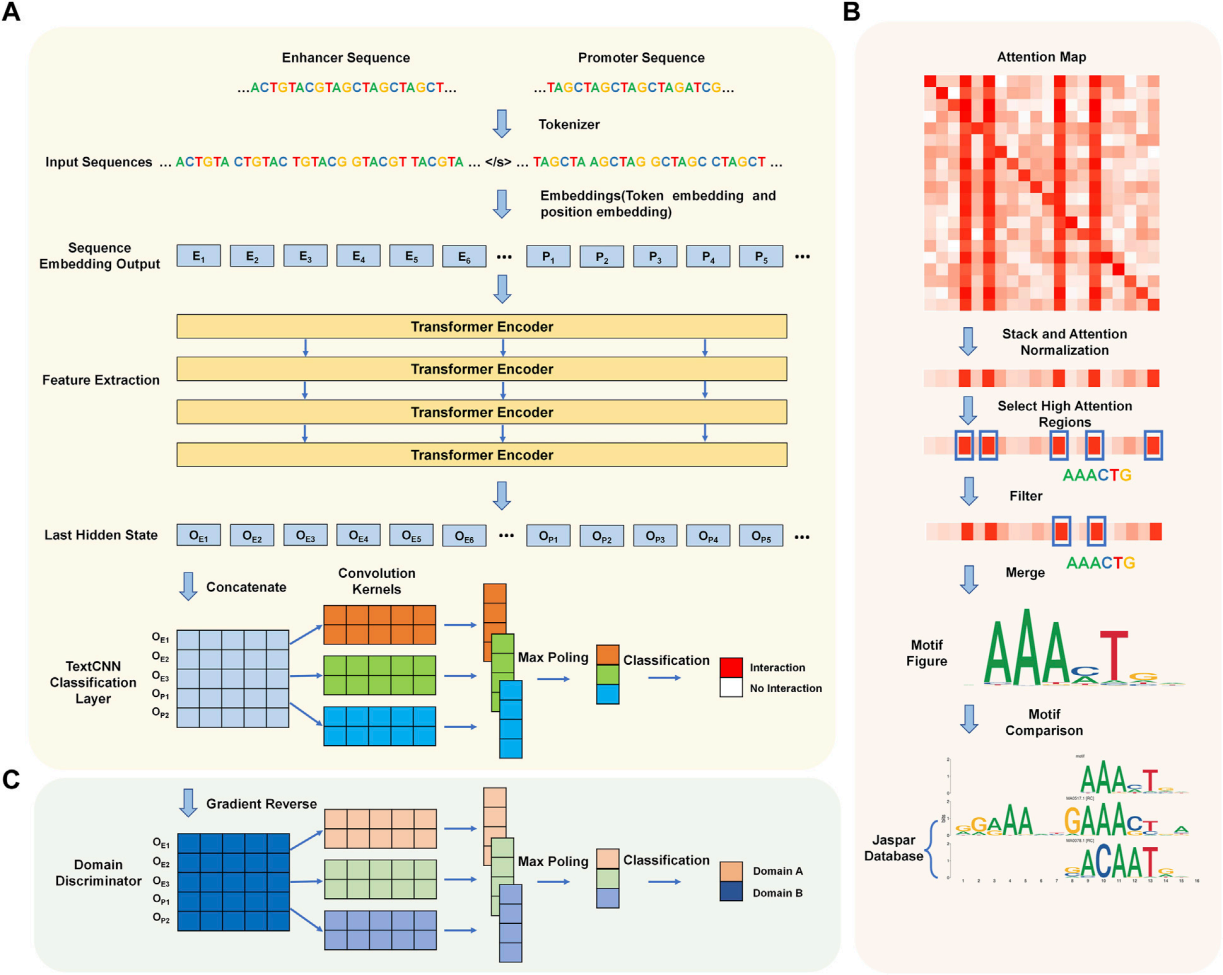
Workflow of TF-EPI. **(A)** Cell type-specific EPI detection network structure. Generally, it includes four steps: tokenization, sequence embedding, feature extraction and classification. **(B)** The process of *de novo* motif discovery. **(C)** Model expansion for cross-cell type EPI detection. The Domain Discriminator is used during the model training process to determine whether the input data comes from the source cell line or the target cell line.

We introduce a new predictive model that utilizes Transformer encoders and TextCNN, allowing for the automatic prediction of EPIs based solely on their DNA sequences. The network flow of the algorithm is illustrated in Figure 1A. The entire network flow includes four components: tokenizer, sequence embedding tool, feature extractor, and classifier. In the first step, the DNA sequences of the paired enhancer and promoter are split into 6-mers, also referred to as tokens. We built a dictionary based on different 6-mers and tokenized each enhancer-promoter pair based on the dictionary. Subsequently, two embedding strategies (token embedding and position embedding) are employed to produce a token-based embedding output. The embedding output is fed into four Transformer encoder layers to extract features representing the initial tokens. Finally, the learned features are stacked and inputted into a convolutional neural network for sentence classification, where the classification results indicate whether each enhancer and promoter pair interact.

### 3.2 TF-EPI outperforms state-of-the-art methods on multiple datasets

To assess the accuracy of our model and ensure a fair comparison with other approaches, we conducted a comparative analysis of our model against the EPIANN and EPI-DLMH methods, which use only DNA sequences across the BENGI and K562 cell line of the Fulco datasets.

**FIGURE 2:**
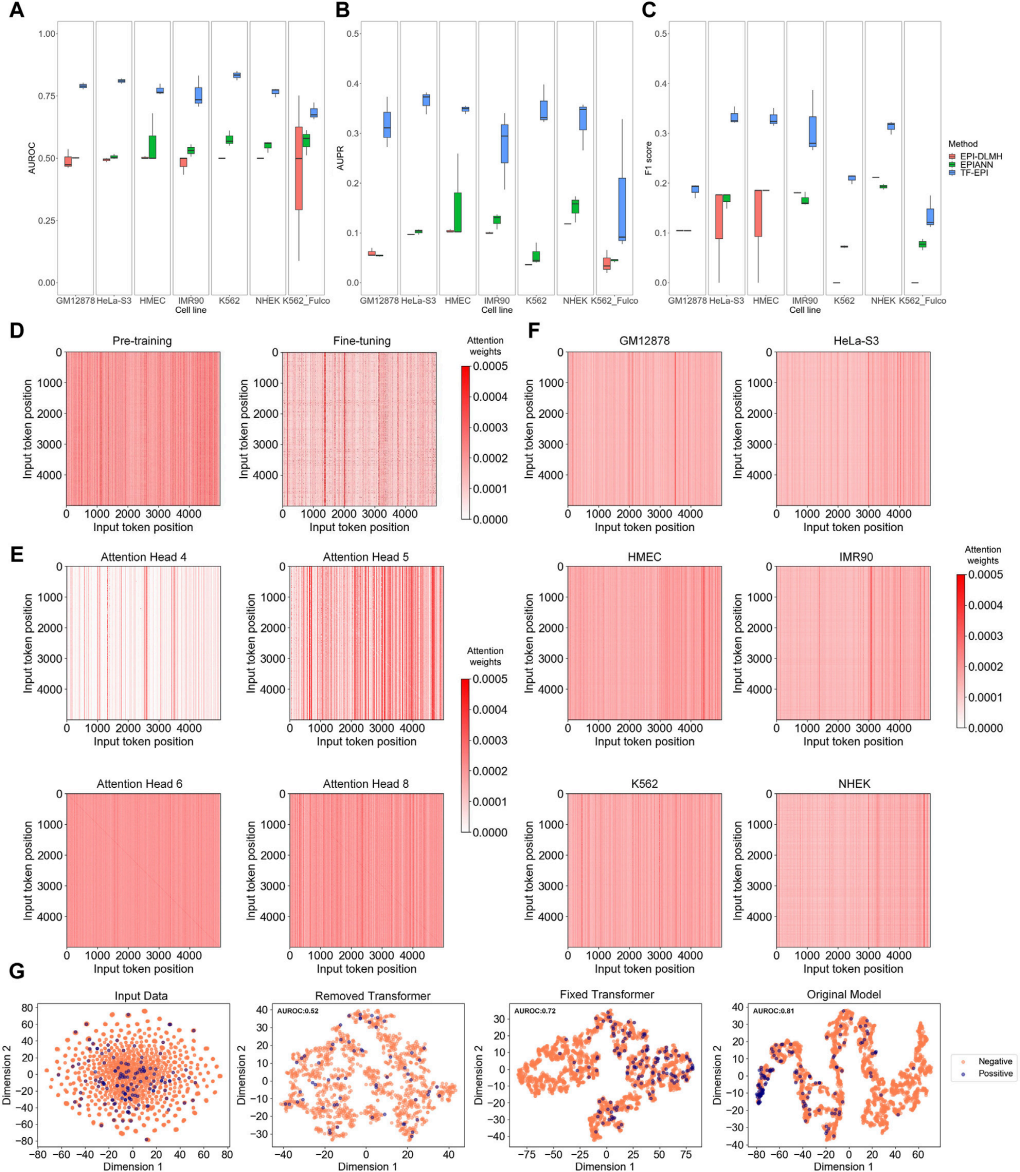
Model comparison and analysis on Transformer and Transformer attention. **(A–C)** Model comparison on multiple benchmark cell lines. Each box represents the results of three independent tests. From left to right, they are respectively AUROC (Area Under the Receiver Operating Characteristic Curve), AUPR (Area Under the Precision-Recall Curve), and F1-score of each cell line. **(D–F)** Attention analysis of Transformer encoder. In each attention matrix, position 0 represents the “CLS” special token, positions 1 to 2,995 represent the 6-mers of the enhancer sequence, 2,996 is the “SEP” special token, and positions 2,997 to 4,991 represent the 6-mers of the promoter sequence. **(D)** Comparison of attention matrix between pre-trained and fine-tuned models. **(E)** Comparison of attention matrices of different attention heads. **(F)** Average attention matrix of different cell lines. **(G)** T-SNE visualization results. The figures, from left to right, respectively show the T-SNE visualization of the tokenized input data, the embedding output of the model trained without Transformer encoder layers, the embedding output of the model trained with fixed Transformer encoder layers (let the parameters maintain the values of pre-trained model), and the embedding output of model from normal training.

TF-EPI achieved the highest AUROCs on all cell lines compared to the other methods (Figures 2A–C). The performance of TF-EPI was particularly outstanding with the BENGI dataset. Although the other two methods reported high accuracy with the TargetFinder dataset, these high performances may be due to the presence of several highly similar low-dimensional features in many enhancer-promoter pairs in the TargetFinder dataset (Xi and Beer, 2018; Moore et al., 2020). Our results confirmed this, particularly for the BENGI dataset, where the other two sequence-based methods achieved approximately, an average AUROC of only 0.5 and average AUPR of only 0.1, indicating their ineffectiveness in learning EPI-related knowledge solely from sequences in this dataset. In contrast, our method, which utilizes powerful deep learning architecture Transformer, effectively learns high-dimensional sequence interactions, thereby achieving more accurate EPI classification results. With the K562 cell line of the Fulco dataset, the performances of all three methods were relatively poor, which may be due to the small sample size. However, our method maintained a higher accuracy than the other two methods, demonstrating its superior ability to extract information from DNA sequences.

We trained these models on one NVIDIA Tesla A100 40G GPU and recorded their runtimes on training sets of varying sizes. Specifically, on the BENGI GM12878 dataset, the training times for the three methods were: 39 h (TF-EPI), 115 h (EPIANN), and 24 h (EPI-DLMH). On the relatively smaller Fulco dataset, the training times were: 2.9 h (TF-EPI), 8.3 h (EPIANN), and 1.8 h (EPI-DLMH). This also demonstrates that our method can achieve much more accurate EPI classification results within a comparable time.

### 3.3 Transformer encoders improve accuracy and help capture diverse information

To investigate why TF-EPI improved the predictive performance, we first compared the attention matrices of TF-EPI acquired after pre-training and after fine-tuning. The attention heatmaps were used to visualize the attention weights, where the color intensity of each cell represents the model focus on different positions within the input sequence, with redder colors indicating higher attention weights. We randomly sampled interacting one enhancer-promoter pair from the GM12878 cell line dataset and input it into the pre-trained and fine-tuned models to observe differences in the attention matrix of the last Transformer encoder layer. As shown in Figure 2D, the pre-trained model exhibits more strong vertical lines, with the values within each line being roughly the same. However, in the fine-tuned model, not only are these strong vertical lines fewer, but the values within each line do not tend to be the same. This suggests that the attention distribution of the pre-trained model was more general, whereas that of the fine-tuned model focused more on detailed areas. This result reflects the differences between the attention matrices obtained from pre-trained model and the matrices after fine-tuning.

To confirm whether the attention heads focused on different regions within the enhancer and promoter sequences, we used enhancer-promoter pairs from the K562 cell line and calculate the average attention matrix of each attention head from the last Transformer encoder layer for visualization (Figure 2E). The lines on the diagonals of some attention heads indicate that these attention heads focus on local interactions. Additionally, there were many vertical lines with high attention, suggesting that at these positions, the corresponding 6-mers were highly correlated with all other 6-mers, indicating their significant role in influencing the embedding output. Different attention heads capture different types of local and global information, which are then generally weighted and integrated. This allows our model to acquire diverse dimensional sequence features, with better identification of potential high-dimensional interactions between sequences, ultimately leading to improved EPI classification.

Moreover, to evaluate which part of the input DNA sequences was the most focused, we input all training samples from each cell line into the corresponding trained models. We then obtained the attention matrices from the last Transformer encoder layer for each sample and averaged these matrices (Figure 2F). Interestingly, for most models, there are some strong vertical lines around position 2,996 (the position of “SEP” token). These vertical lines indicate that the models assign higher attention weights around the “SEP” token, meaning these positions play an important role in the model’s processing and understanding of the input data, which in turn highlights the significance of the “SEP” token in separating the input data and aiding the classification of EPIs. In addition, the models focused more on certain regions at the ends of enhancers and promoters. This implies that we captured information from relatively distant regions, which aided in the classification of EPIs.

Afterward, to examine the effect of the Transformer on our model, we removed the Transformer part of our model, or fixed the parameters of Transformer encoders and trained only the TextCNN component. These approaches, compared with our original model, were analyzed using t-SNE for sequence visualization. The results for the GM12878 (Figure 2G) and other cell lines (Supplementary Figure S1) showed that both removing the Transformer encoder layers and fixing the Transformer encoder parameters led to disorganized embedding outputs. In contrast, the training of the Transformer and TextCNN together, yielded more distinct separations between the positive and negative samples. These results imply the importance of the Transformer encoders and show that the TextCNN model alone may not be sufficient to achieve good classification results. Collectively, the introduction of the Transformer and its attention mechanism helped our model learn the multi-dimensional features of DNA sequences and assisted in the classification of EPIs.

### 3.4 TF-EPI discovers biologically meaningful motifs

**FIGURE 3:**
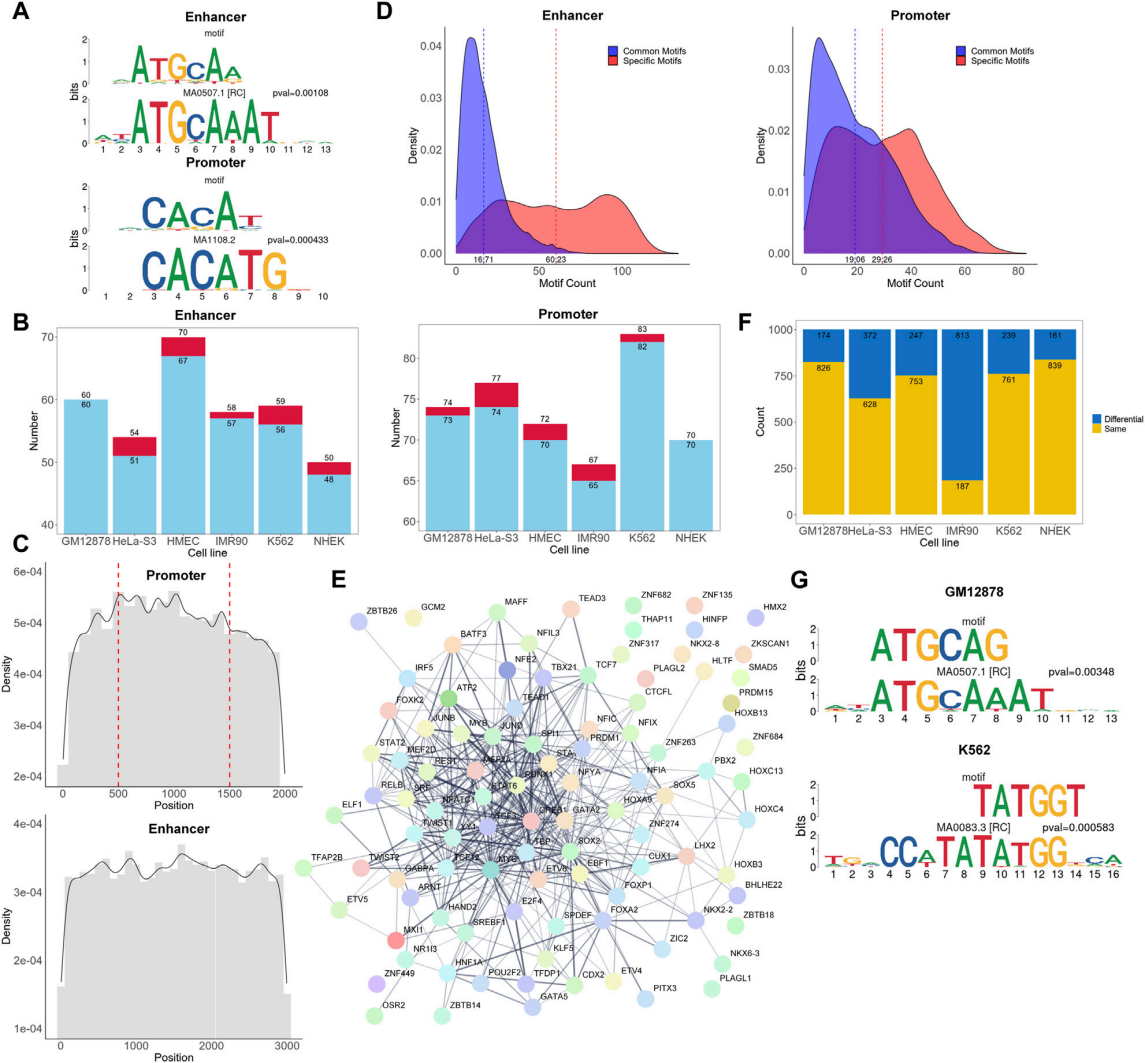
Analysis of motifs and corresponding TFs. **(A)** Examples of *de novo* motifs identified and similar known motifs recorded in the JASPAR database. The *p*-values are calculated using Tomtom. **(B)** Overall number of *de novo* motifs discovered and those with similar counterparts in the JASPAR database. Each red bar and the upper value represent the number of discovered *de novo* motifs, while each blue bar and the lower value represents the number of *de novo* motifs that are highly similar to known motifs in the JASPAR database. **(C)** Density plot of the occurrence frequency of high attention sequences at different positions in the enhancers and promoters. The position 500 and 1,500 of the promoter sequence represents the transcription start site. **(D)** Density plot of significant attention region counts of each promoter and enhancer sequence. The dashed lines represent the average number of common motifs and specific motifs in each sequence. **(E)** The protein-protein interaction network of TFs found in cell line GM12878. **(F)** Number of same and different 6-mer pairs between positive and negative samples for each cell type. **(G)** Most frequently occurring differential 6-mers in cell lines GM12878 and K562.

Many existing models have elaborated the importance of CTCF and cohesin for promoter-enhancer interactions (Xi and Beer, 2021; Gschwind et al., 2023; Luo et al., 2023). Here, we aim to investigate whether there are other subsequences in more extensive interacting chromatin regions that can simultaneously affect the promoter-enhancer interaction process. Hence, we explored the biologically significant information contained within the attention matrices, investigated which regions in the attention matrices of the positive samples were more influential in classification, and discovered *de novo* motif sequences using the process shown in Figure 1B. One example motif each from the enhancer and promoter regions of GM12878 are shown in Figure 3A. Each figure shows a comparison of the discovered motifs with known human motifs in the JASPAR database using Tomtom. The results indicated that over 95% of the *de novo* motifs were highly similar (*p* < 0.02) to known motifs in the JASPAR database (Figure 3B; Supplementary Table S2), which validates the ability of our approach to learn conserved sequential characteristics while focusing on the TF regions in the sequences, thus acquiring biologically meaningful information.

In addition, we matched the cell line-specific TFs corresponding to the motifs identified against the UniBind database to check if they were consistent with TFs in a known database (Supplementary Table S2). Because the database has fewer TF records for HMEC, IMR90, and NHEK cell lines, with only 1, 14, and 1 TFs, respectively, we mainly focused on the other 3 cell lines. In the GM12878, HeLa-S3, and K562 cell lines, we identified 25, 10, and 31 TFs in the promoter regions, respectively, and 19, 11, and 31 TFs in the enhancer regions, respectively, as reported in the UniBind database (Supplementary Table S2). This strongly suggests that the identified TFs are likely to be active and important in their respective cell lines, further substantiating the biological significance of the identified motifs. TFs that have not been documented may also be important for specific cell types.

We further examined whether it was possible to find correlations between the discovered TFs and source information for each cell line. From the IMR90 cell line, we identified TFs such as FOXA2 and SOX2. FOXA2 has been proved to be important for lung morphogenesis, and SOX2 is crucial for the early stages of lung development (Wan et al., 2004; Eenjes et al., 2022). In the HMEC cell line, we found TFs such as STAT5 and FOXA1. STAT5 can be activated through various signaling pathways in mammary epithelial cells, and FOXA1 is crucial for mammary gland development (Furth et al., 2011; Seachrist et al., 2021). These findings demonstrate the capability of our method to identify TFs relevant to the source information of the cell line.

To understand the functional significance of different regions within promoters and enhancers in EPIs, we calculated the frequency of occurrences of high attention motifs in various sequence positions (Figure 3C). In promoter regions, the frequency of motif occurrences reaches a maximum around multiple positions, such as the transcription start site, and generally shows a declining trend towards both ends. Similarly, in the enhancer regions, the frequency is higher at the center than on the sides. This indicates that there may be core regions or sites within the sequence similar to the TSS that play an important role in the regulation of gene expression. In addition, increasing the sequence length may be beneficial for capturing more meaningful information.

To explore the potential “biological language grammars” of enhancers or promoters, we analyzed the significant attention region counts of all positive EPIs (Figure 3D). We categorized these significant attention regions into two types: common motifs, which are similar motifs found across multiple positive sequences; and specific motifs, which refer to those motifs that have relatively insufficient representation in the positive samples. These specific motifs either had low counts in positive samples or failed to show significant enrichment in positive versus negative samples. Interestingly, in both enhancer and promoter regions, the number of specific motifs per sequence generally exceeded that of common motifs. This suggests that a combination of common and specific motifs may play an important role in determining potential EPIs. Detailed information of these common motifs can be found on our GitHub repository under “https://github.com/lbw1995/TF-EPI-supplementary-data/blob/main/Supplementary_Results.ar.gz.”

Furthermore, we used the STRING (Szklarczyk et al., 2023) database to investigate whether the TFs in the enhancer and promoter regions interacted with each other. We present the network of interactions between TFs found in the cell line GM12878 in Figure 3E and the networks of other cell lines in Supplementary Figure S2. Figure 3E shows that there are 436 interactions among the TFs with interaction scores higher than 0.4. Additionally, we randomly selected an equal number of TFs from the complete set of known human TFs and input them into the STRING database. On average, these random selections yielded about 58 interactions with scores higher than 0.4 (Supplementary Figure S3), significantly less than the 436 interactions we observed. This finding suggests that these biologically significant and interacting TFs are likely the key reasons for the excellent performance of TF-EPI with the BENGI dataset.

### 3.5 TF-EPI discovers many cell-type specific k-mer interactions

To further investigate whether there were inconsistencies in the interactions between k-mers in the positive and negative samples, we separately calculated the frequency of different 6-mer pairs with higher attention values for both the positive and negative samples in each cell line. We then examined the top thousand most frequently occurring high attention 6-mer pairs to identify those that appeared in both positive and negative samples and were exclusive to the positive samples. These 6-mer pairs, which only appeared in the positive samples, could also have a significant impact on EPI. These 6-mer pairs are listed in Supplementary Table S3, and their number counts are detailed in Figure 3F. Additionally, we arranged the occurrence frequency of these 6-mers and examined whether high-frequency 6-mers were associated with important TFs. We also used Tomtom to identify motifs related to these 6-mers and checked whether the motifs were recorded in the UniBind database.

Consequently, we found that the TFs corresponding to the most frequently occurring 6-mers, such as POU2F2 and SRF, are important for specific cell lines and are recorded in the UniBind database (Figure 3G). Moreover, the TF POU2F2 corresponding to the most frequently occurring 6-mer in GM12878 cell line was included in our discovered *de novo* motifs, while the TF SRF for K562 cell line was not. This highlights that these two analytical strategies—searching for short sequences in one-dimensional and two-dimensional attention regions—can uncover both same and different important TFs for specific cell lines. In summary, through in-depth analysis of the attention matrix, we revealed the potential importance of 6-mer pairs that appeared only in positive samples and demonstrated the connection between these 6-mer pairs and key TFs.

### 3.6 TF-EPI reveals heterogeneity of EPIs among different cell lines

To test whether the motifs in the enhancer and promoter regions of different cell lines corresponded with consistent TFs, we first consolidated the identified TFs and examined whether there was consistency between TFs found in enhancer regions and those in promoter regions. The results indicated that the majority of TFs were unique to either the enhancer or promoter region, with a small portion present in both (Supplementary Figure S4). These unique TFs suggest that enhancers and promoters require distinct regulatory mechanisms to initiate or enhance transcription effectively. In addition, both enhancer and promoter regions can be recognized and bound by some consistent TFs, thereby collaboratively regulating gene expression.

**FIGURE 4:**
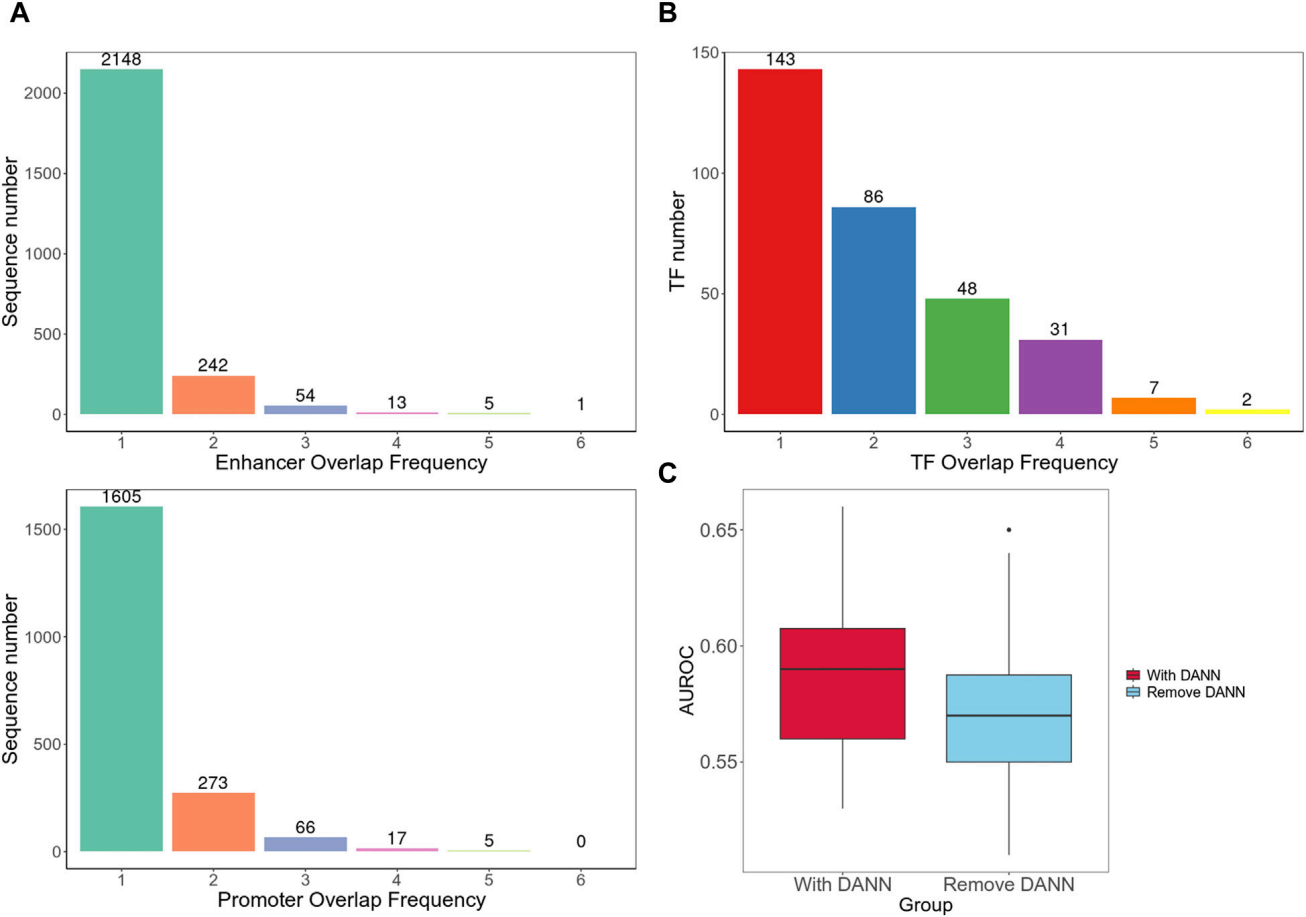
Cross-cell line analysis. **(A)** Number of overlapping sequences among different cell line datasets. **(B)** Frequency of occurrence of TFs in different cell types. **(C)** Cross-cell line EPI detection performance. It contains two group of AUROC values; the left box plot is for testing on the target dataset with model including DANN and trained on both target and source datasets; the right box plot is for testing on the target dataset using model trained only on the source dataset without DANN.

Furthermore, we separately integrated all enhancer and promoter sequences of different cell lines and used BEDTools to calculate the number of overlapping sequences among different cell line datasets (Quinlan and Hall, 2010). The results indicated a relatively low degree of overlap between different cell lines (Figure 4A). Next, we compared all the TFs from the enhancer and promoter regions found in each cell line and counted the number of TFs that appeared one to six times (Figure 4B). We observed that the majority of TFs existed in only one or 2 cell types, which validated the high heterogeneity of EPIs in cell lines.

Interestingly, we also found some shared characteristics of EPIs among different cell lines. The transcription factor MXI1, which was widely focused by the model in all 6 cell lines, plays a significant role in apoptosis and differentiation (Schreiber-Agus et al., 1998; Benson et al., 1999). The duality of our findings illustrates both the diversity and commonalities of EPIs among different cell lines, demonstrating the ability of our model to capture complex gene regulation patterns.

In addition, we introduced a method, as shown in Figure 1C, to achieve cross-cell line EPI detection. We used each cell line in the BENGI dataset as a training set and then used data from every other cell line as test set. We initially incorporated the DANN structure trained with both the source and target datasets. We then trained the model solely using the source dataset, excluding the target domain data and DANN structure. The model performances with the DANN structure were better. Models with DANN were able to increase the average AUROC of prediction results on target cell lines by 0.015, compared to those without the DANN (Figure 4C; Supplementary Table S4). This indicates that although the high heterogeneity of EPIs between cell lines limits the model performance significantly, our approach still improves detection accuracy to a certain extent.

Taken together, our study not only confirms the high heterogeneity of EPIs among different cell lines, but also demonstrates the effectiveness of our model in identifying and understanding EPIs across different cell lines.

## 4 Discussion

Our study introduces TF-EPI, a Transformer-based model, marking a significant step forward in the prediction and understanding of EPIs. The application of the Transformer architecture in TF-EPI, particularly its attention mechanism, allowed for an intricate understanding of which sub-sequences significantly contribute to the classification results. This approach facilitated the discovery of biologically meaningful motifs and short sequence pairs that are crucial for EPIs, offering insights into the regulatory elements of gene expression.

The comprehensive analysis across various cell lines clearly demonstrates the superiority of TF-EPI over the existing state-of-the-art methods, particularly in leveraging deep learning to navigate the complex landscape of genomic interactions. Notably, our model identified motifs of TFs corresponding with known motifs in databases like JASPAR and UniBind. This not only corroborates the validity of our predictions but also suggests its potential utility in capturing the specific and common “biological language grammars” potentially involved in the progress of gene regulation.

Remarkably, we found that EPIs of different cell types are highly heterogeneous. This heterogeneity, as evidenced by the varied TFs identified in different cell lines, underscores the complexity of gene regulation mechanisms. Despite this diversity, our model successfully identified MXI1 across all 6 cell lines, a TF known for its role in cell apoptosis and differentiation. This result highlights commonalities in EPI across different cell types and attests to the capability of TF-EPI in discerning critical motifs that are pivotal for multiple cell types.

Despite the intrinsic heterogeneity in EPIs across different cell lines, TF-EPI achieved a certain improvement in detection accuracy in the cross-cell line EPI detection. This advancement demonstrates the potential of transfer learning in overcoming the challenges posed by cell-specific gene regulation and underscores the necessity of versatile models in the dynamic field of genomics.

In conclusion, TF-EPI effectively handles the complexities of genomic data, offering valuable insights into gene regulation and interaction. Despite the challenges posed by the heterogeneity of EPIs, the adaptability and precision of our model underscore its utility in exploring cell-specific regulatory networks, thereby contributing to advanced research in gene regulation.

## 5 Limitations and future work

Although TF-EPI shows higher performance on multiple cell line datasets than some other DNA sequence based methods, its complex model structure increases the time and memory usage. Besides, the information obtained solely from DNA sequences is limited, which may result in the accuracy of TF-EPI being inferior to methods based on epigenomic features. We plan to introduce a more lightweight transformer model and utilize a broader array of features, including those derived from DNA sequences and selected epigenomic features, to enhance the accuracy of EPI predictions further and attempt to identify the connections between DNA sequences and these epigenomic features. In addition, we would use more higher resolution EP interaction data to further validate the consistency of our predictions with rigorous experimental verification results (Yao et al., 2024).

## Data Availability

All original data used in this study were obtained from public resources and can be downloaded from original publications, as mentioned in Materials and methods section. The codes and processed data presented in the study are deposited in the GitHub repository at https://github.com/lbw1995/TF-EPI.
