# Supplementary material for: TF-EPI: an interpretable enhancer-promoter interaction detection method based on Transformer
Source: Front Genet. 2024 Aug 9;15:1444459. doi: 10.3389/fgene.2024.1444459 (PMC11341371; doi:10.3389/fgene.2024.1444459)
Supplement: Supplementary file 2 [file DataSheet1.pdf]

Contents

Supplementary Notes 2

Supplementary Note 1: Description of the datasets 2

Supplementary Note 2: Detailed model parameters and model training of TF-EPI.................2

Supplementary Note 3: Attention mechanism of Transformer and the processing of attention matrix 4

Supplementary Note 4: Details of motif discovery 5

Supplementary Note 5: Details of cross-cell line detection of EPIs 5

Supplementary Note 6: Evaluation metrics..6

Supplementary Figures 7

Supplementary Figure 1: T-SNE visualization of input data, embedding output of network without Transformer, embedding output of network with fixed Transformer and embedding output of original network...7

Supplementary Figure 2: TF interaction networks of different cell lines.................................8

Supplementary Figure 3: Random TF interaction networks 9

Supplementary Figure 4: TFs comparison between promoter regions and enhancer regions.10

Supplementary Figure 5: Model comparison based on datasets split by chromosomes 11

Supplementary Tables 12

Supplementary Table 1: Number of samples for BENGI and Fulco dataset..........................12

Supplementary Table 2: TF consistency analysis results with JASPAR and Unibind database 12

Supplementary Table 3: List of high attention 6-mer pairs exclusive to positive samples 12

Supplementary Table 4: Performance of cross-cell line EPI detection 12

References..13

Supplementary Notes

Supplementary Note 1: Description of the datasets

In our paper, we used two distinct datasets as benchmark datasets and tested them with various methods. The first dataset is the BENGI dataset. For this, we adopted the same data processing method as TransEPI. The dataset's genes were mapped to transcripts using GENCODE annotation, thus converting enhancer-gene pairs into EP pairs. Here, promoters were defined as 1500 bp upstream and 500 bp downstream of the transcript start site (TSS). Positive samples were annotated through 3C sequencing results, and negative samples were generated by pairing enhancers with non-interacting genes within the 95th percentile of the enhancer-gene distances of positive samples.

The second dataset is from the paper by Joseph Nasser et al. In Supplementary Table 5 of their paper, they presented CRISPR experimental validation results for some enhancer-gene predictions. We also defined promoters as 1500 bp upstream and 500 bp downstream of the TSS. Since the dataset had sufficient positive and negative samples only for the K562 cell line, we selected K562 cell line data with 'IncludeInModel' values equals to TRUE as a new dataset for comparative experiments between different methods. The label is annotated by the row 'Significant'.

For these datasets, we used the starting position and ending position of each enhancer and promoter, and according to the human reference genome hg19, we extracted their DNA sequences for subsequent method comparisons and analyses. The number of samples for each cell line in different datasets is presented in Supplementary Table 1. For each cell line in BENGI dataset, the training set contained 90% of the data, validation set contained 5% of the data and the test set contained the remaining 5%. For K562 cell line in the Fulco dataset, due to its relatively small total data amount, we set the training set contained 80% of the data, validation set contained 10% of the data and the test set contained the remaining 10%.

Supplementary Note 2: Detailed model parameters and model training of TF-EPI

For our model, we used the transformers library provided by HuggingFace to build our Transformer network. Each layer of the Transformer encoder includes 12 attention heads, with each hidden layer having an output size of 768, which has been widely used on multiple NLP tasks. Given that the

length of the enhancer sequences is 3000 and the promoter sequences is 2000, we set the max token length of the Transformer to 5100 to ensure it can accommodate all enhancer tokens and promoter tokens. We concatenated the embedding results of each token from the last layer of the Transformer encoder into a matrix whose shape is 5100 multiply 768. This means that each token from original 5100 input tokens is regenerated to a dimension of 768, which was then input into the TextCNN. For the TextCNN, we used three convolutional kernels of size 6 to further extract features from the last layer of the Transformer encoder. We then used max pooling to reduce the feature dimension before feeding the data into a fully connected neural network for EPI detection. During both the pre-training and fine-tuning phases, we used AdamW as the optimizer for model parameters, and calculated the loss function using cross-entropy. The hyperparameters of the fine-tuned models are selected by each validation set of each cell line. Afterwards, we use these hyperparameters to test the model performance on the test dataset.

Additionally, in the model pre-training progress, we randomly generated non-overlapping sequences no longer than 5000bp in length from human reference genome hg19 and converted them into 6-mers. We only performed masked token prediction during the pre-training process. We selected a random starting position and continuously masked 15% of the tokens to enable the model to better learn the long-range dependencies between k-mers in DNA sequences. The loss during training is computed based on the discrepancy between the model's predictions at masked positions and the actual ground truth labels. After 3 epochs in the pre-training process, we obtained the final pre-trained model. Based on this model, we performed subsequent fine-tuning on different cell line datasets. To reduce the risk of overfitting, we used a learning rate warm-up method, where in the first 5% of the total training steps, the learning rate was increased from a lower bound to its maximum and then gradually decreased in the later stages, ultimately yielding the final fine-tuned neural network.

During the fine-tuning process of the model, since the other two comparative methods also adopted the same data augmentation method, which is:

1. Start from the imbalanced data D.
2. Split D into a training set D_{train} by stratified sampling.
3. Augment D_{train} to get a balanced dataset D_{aug}.
4. Train the model on D_{aug}.

In step 3, D_{aug} involves generating new positive samples by randomly shifting each positive promoter and enhancer by a certain distance, thereby creating new enhancer-promoter pairs. We adopted this data augmentation method for a fair comparison. Meanwhile, to evaluate the results of our method under different data partitioning strategies and to assess the impact of overfitting on our model, we adopted data splitting strategy same as that used in the paper of Xi and Beer [2], ensuring that all E-P pairs on each chromosome are in the same test set. We split the NHEK dataset into three new train-validation-test sets following this approach, with each test set only including EP pairs from one chromosome that has a sufficient number of samples. The comparison results of our method with the other methods on these newly split datasets are shown in supplementary figure 5. Although the performance of our method has declined to some extent, it still outperforms the other two methods. This indicates that while our method cannot completely solve the overfitting problem, it still performs better than the other two methods. We have uploaded all the split datasets used in this paper to the following GitHub repository link: https://github.com/lbw1995/TF-EPI-supplementary-data/blob/main/data.tar.gz.

Supplementary Note 3: Attention mechanism of Transformer and the processing of attention matrix

Since most of our analyses are based on the attention matrix, understanding the attention mechanism of the Transformer is very important. The strength of the Transformer comes from its attention mechanism:

$$\text{Attention}(Q, K, V) = softmax\left(\frac{QK^T}{\sqrt{d_k}}\right)V$$

Among it:

$$\begin{cases} Q = XW^Q \\ K = XW^K \\ V = XW^V \end{cases}$$

In our method, X is the input to the Transformer encoder, Q represents the query matrix, K is the key matrix, and V is the value matrix. The attention matrix is calculated as $softmax\left(\frac{QK^T}{\sqrt{d_k}}\right)$, and its size is L by L, where L is the length of the model's input sequence. This matrix reflects the model's distribution of attention weights, showing the focus allocated to other k-mers in the sequence while processing a particular k-mer. This enables us to identify which k-mers the model pays more attention to and to analyze these k-mers.

Since we have introduced a multi-head attention mechanism, for each attention head, there is:

$$Attention_i = Attention(Q_i, K_i, V_i), i = 1, \ldots, h$$

$$Multihead = Concat(Attention_1, \ldots, Attention_h)W^O$$

where, $Attention_i$ corresponds to the attention of each attention head, which is concatenated and then weighted by W^O to obtain the final result.

Additionally, when analyzing the attention matrix, we only focused on the matrix of the last layer of the Transformer encoder, as it most directly influences the model's final output. Furthermore, later Transformer encoder layers can capture more complex and abstract features, thus better reflecting intricate sequence relationships and task-specific information.

Supplementary Note 4: Details of motif discovery

Because our model uses embedding output of all tokens for the classification task, after obtaining the attention matrix of each enhancer-promoter pair, we did not choose to use the attention values between the CLS token and other tokens for subsequent analysis, but instead averaged the attention values of each token with all tokens (including itself) and performed the subsequent analysis. Since we get the attention value of k-mers, we need to convert these attention values to the attention value of each single base. Same as DNABERT, for every single base, we summed and averaged the attention of the k-mers surrounding it and get the attention value for each base. We normalized these attention values to a range of 0 to 1 using min-max normalization. Following this, we set a threshold and iterated to find continuous subregions where the attention exceeded this threshold and filtered these subregions using a hypergeometric test. After obtaining these filtered subsequences, we used sequence alignment algorithms to compare query motifs to see if they could be merged, ultimately obtaining the merged motif sequences. When using Tomtom, we set the parameters of Tomtom as default values and compare our *de novo* motifs with known motifs in JASPAR database.

Supplementary Note 5: Details of cross-cell line detection of EPIs

We used one training set of each cell line to conduct cross-cell line detection of EPIs. Since each training dataset is label-imbalanced and varies in the number of sequences, we employed downsampling to randomly select negative samples until achieving a total of 5000 samples. This approach resolves the imbalance and facilitates subsequent cross-cell line training. In addition, due to an increase in model parameters from integrating the domain discrimination component, we incorporated sparse self-attention mechanism of Longformer [1] to reduce the demands on memory.

Specifically, we used an attention window of size 100 in the first three layers of the transformer encoder, and a full-size attention window of 5100 in the fourth layer. This approach helps reduce the computational resource consumption while maximizing information capture.

During training, we fed both source and target cell lines into the network. For the EPIs classification task, we updated the parameters only based on the source cell line. Meanwhile, for the domain discrimination task, we updated the parameters based on both the source and target cell lines. During testing, we assessed the model only in the target cell line and determined whether the enhancer-promoter pairs interacted.

Supplementary Note 6: Evaluation metrics

For the fine-tuning tasks, performance was evaluated using the following metrics:

$$Precision = \frac{TP}{TP + FP}$$

$$Recall = \frac{TP}{TP + FN}$$

$$F1 = 2 \times \frac{Precision \times Recall}{Precision + Recall}$$

In addition, we also calculated the area under the receiver operating characteristics curve (AUROC) and/or the area under the precision-recall curve (AUPR).

Supplementary Figures

Supplementary Figure 1: T-SNE visualization of input data, embedding output of network without Transformer, embedding output of network with fixed Transformer and embedding output of original network

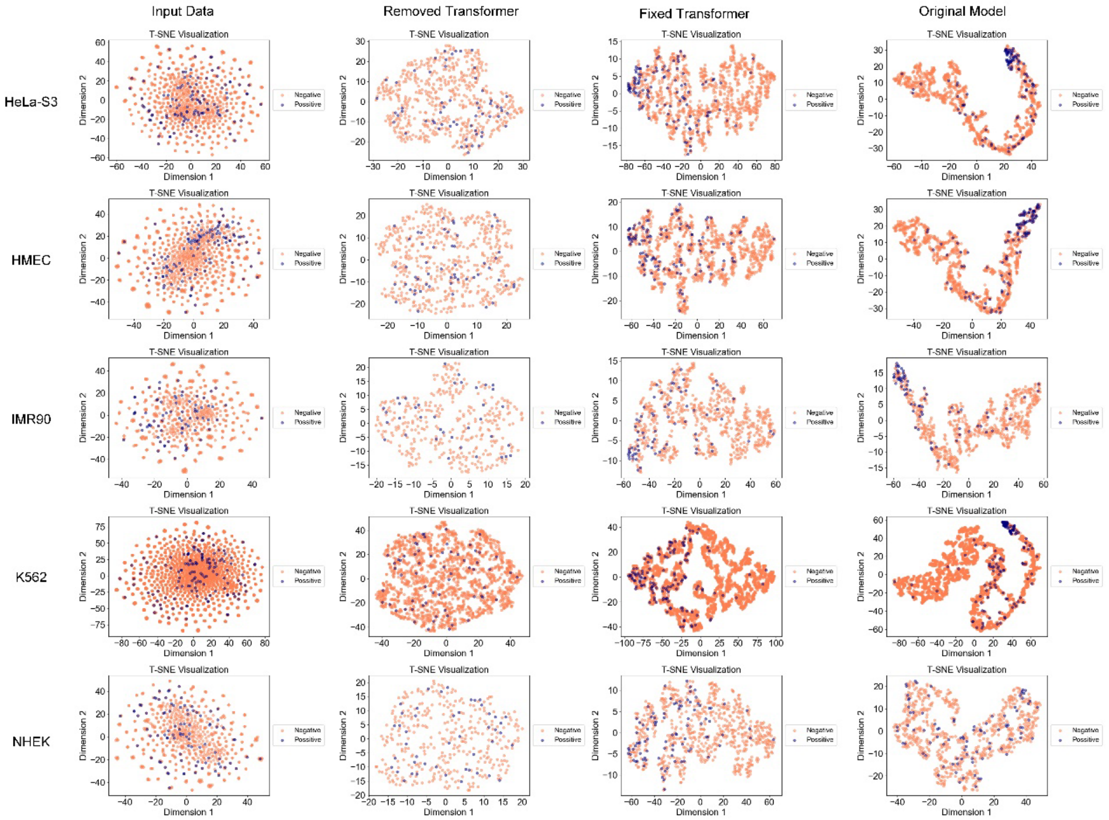

Supplementary Figure 2: TF interaction networks of different cell lines

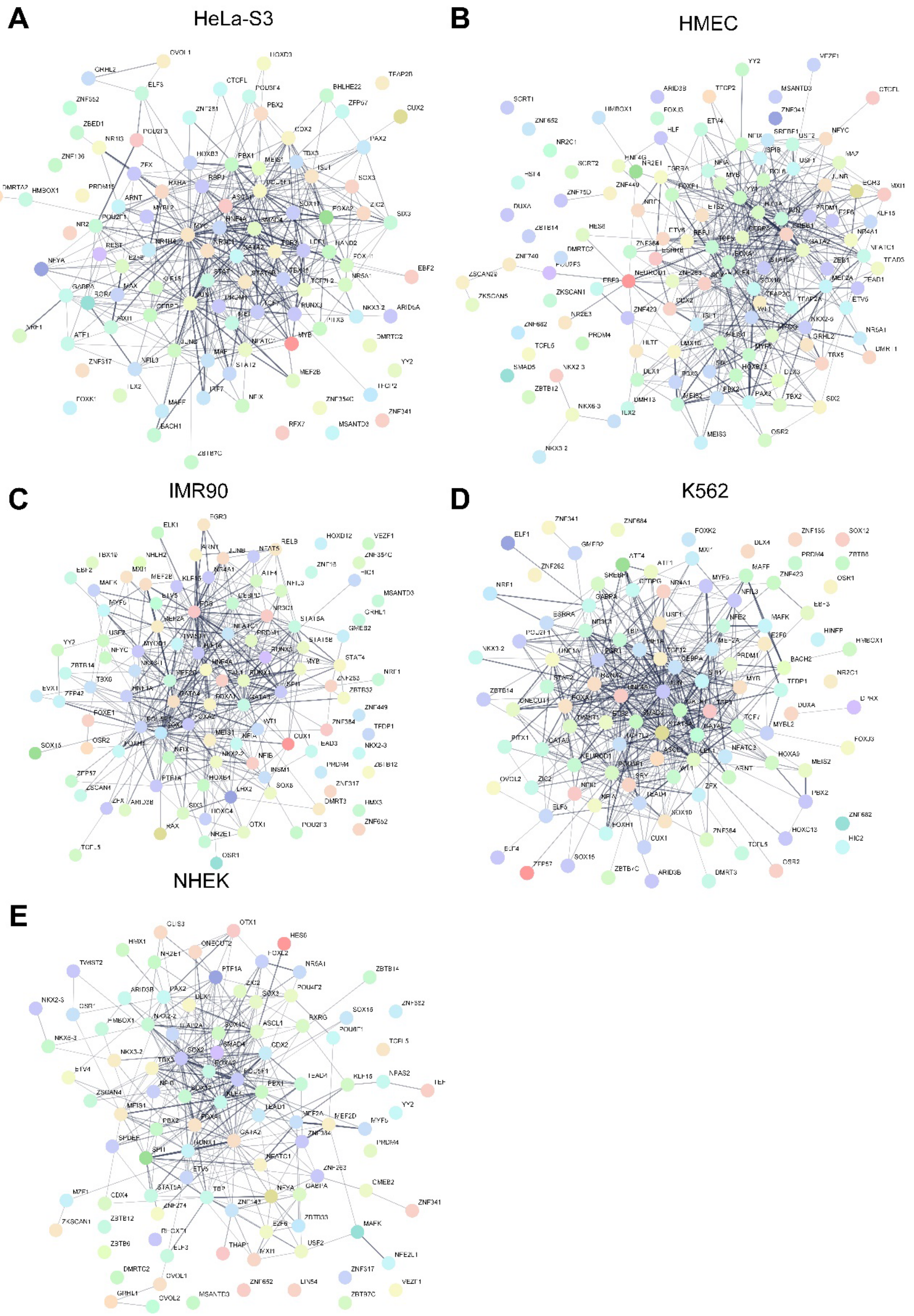

Supplementary Figure 3: Random TF interaction networks

Random TF group1

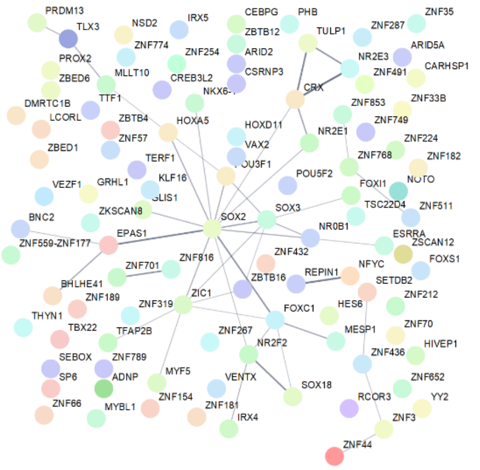

Random TF group2

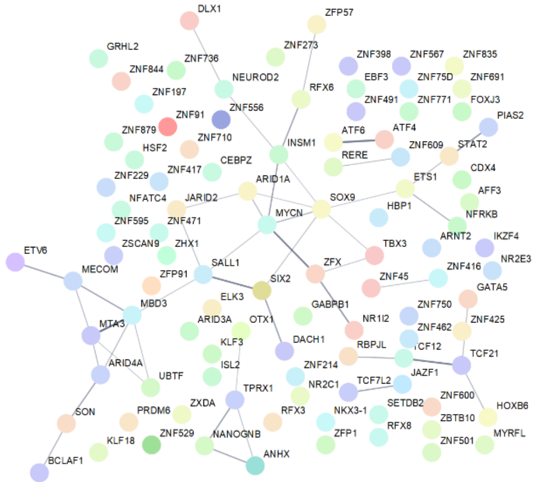

Random TF group3

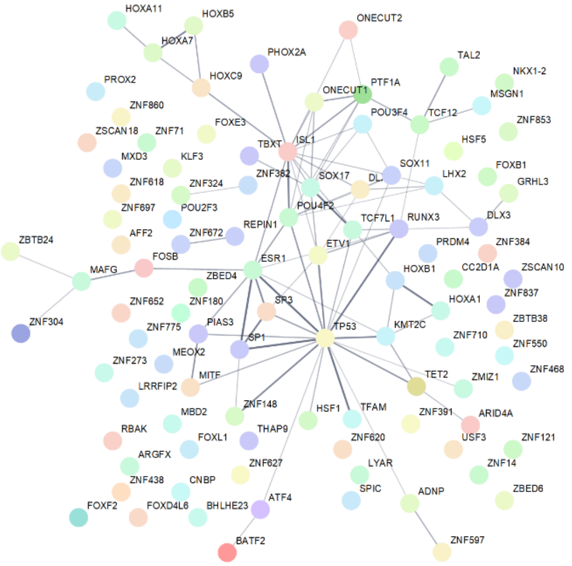

Supplementary Figure 4: TFs comparison between promoter regions and enhancer regions

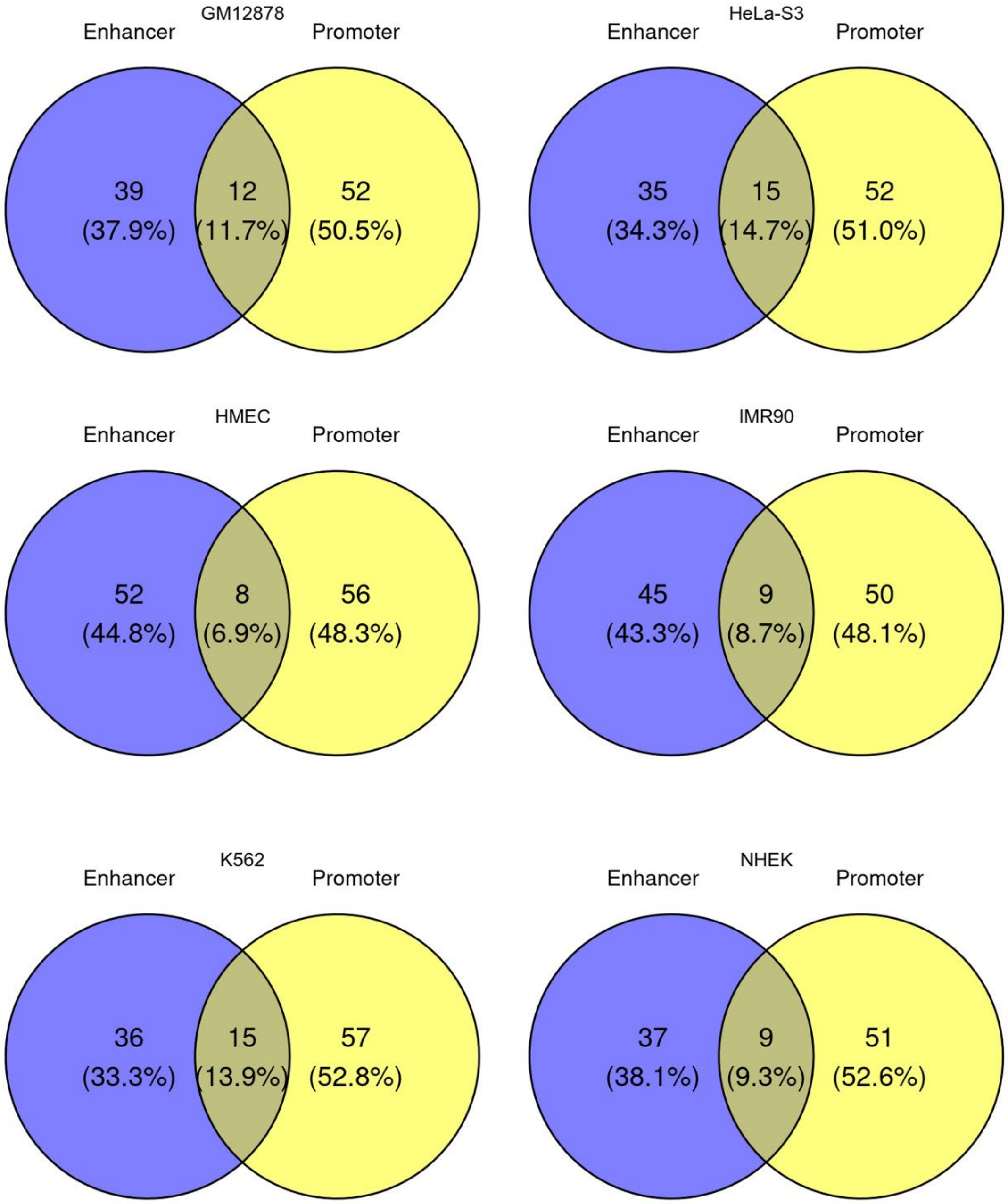

Supplementary Figure 5: Model comparison based on datasets split by chromosomes

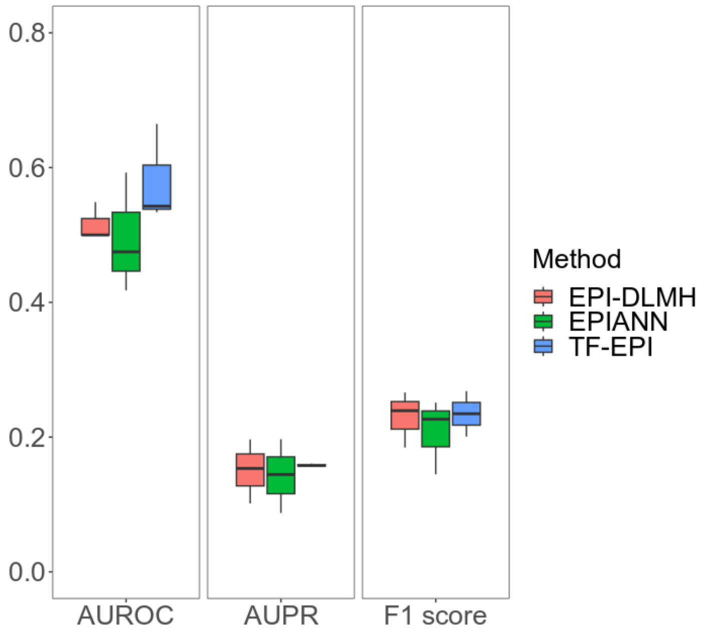

Supplementary Tables

Supplementary Table 1: Number of samples for BENGI and Fulco dataset

Cell line	Positive sample	Negative sample
**GM12878**	2695	46212
**HeLa-S3**	2256	21086
**HMEC**	2286	20019
**IMR90**	1468	13268
**K562**	2765	73299
**NHEK**	1820	13582
**K562_Fulco**	134	3720

Supplementary Table 2: TF consistency analysis results with JASPAR and Unibind database

The results are listed in additional file named "Supplementary Table 2.xlsx".

Supplementary Table 3: List of high attention 6-mer pairs exclusive to positive samples

The results are listed in additional file named "Supplementary Table 3.xlsx".

Supplementary Table 4: Performance of cross-cell line EPI detection

Each table cell shows test results using one cell line as the source dataset (left) and every other cell line as the target dataset (above). The upper table is for testing on the target dataset with model including DANN and trained on both target and source datasets; the lower table is for testing on the target dataset using model trained only on the source dataset without DANN.

Target / Source	GM12878	HeLa-S3	HMEC	IMR90	K562	NHEK
GM12878	NA	0.56	0.62	0.58	0.59	0.57
HeLa-S3	0.53	NA	0.6	0.59	0.53	0.61
HMEC	0.59	0.6	NA	0.66	0.59	0.63
IMR90	0.57	0.61	0.64	NA	0.55	0.61
K562	0.58	0.56	0.57	0.56	NA	0.56
NHEK	0.55	0.6	0.59	0.61	0.55	NA

Target / Source	GM12878	HeLa-S3	HMEC	IMR90	K562	NHEK
GM12878	NA	0.56	0.58	0.59	0.57	0.56
HeLa-S3	0.53	NA	0.55	0.57	0.54	0.59
HMEC	0.56	0.6	NA	0.64	0.59	0.63
IMR90	0.57	0.57	0.65	NA	0.57	0.61
K562	0.54	0.56	0.53	0.54	NA	0.54
NHEK	0.55	0.58	0.57	0.57	0.51	NA

References

[1]. Beltagy I, Peters M E, Cohan A. Longformer: The long-document transformer[J]. arXiv preprint arXiv:2004.05150, 2020.

[2]. Xi W, Beer M A. Local epigenomic state cannot discriminate interacting and non-interacting enhancer–promoter pairs with high accuracy[J]. PLoS computational biology, 2018, 14(12): e1006625.
